# Genomic and Transcriptomic Study for Screening Genes Involved in the Limonene Biotransformation of *Penicillium digitatum* DSM 62840

**DOI:** 10.3389/fmicb.2020.00744

**Published:** 2020-04-22

**Authors:** Lu-Lu Zhang, Wen Huang, Ying-Ying Zhang, Gang Fan, Jin He, Jing-Nan Ren, Zhi Li, Xiao Li, Si-Yi Pan

**Affiliations:** ^1^Key Laboratory of Environment Correlative Dietology, Ministry of Education, College of Food Science and Technology, Huazhong Agricultural University, Wuhan, China; ^2^State Key Laboratory of Agricultural Microbiology, College of Life Science and Technology, Huazhong Agricultural University, Wuhan, China

**Keywords:** *Penicillium digitatum* DSM 62840, limonene biotransformation, genomic, transcriptomic, cytochrome P450

## Abstract

α-Terpineol has been widely used in daily chemical, pharmaceutical, food, and flavor industries due to its pleasant odor with high economic value and pharmacological action. Our previous study showed that *Penicillium digitatum* DSM 62840 was an efficient biocatalyst for the transformation of limonene to α-terpineol. Thus, it was meaningful to explore the genome features and the gene expression differences of strain DSM 62840 during limonene biotransformation, and the detailed bioconversion pathways. In this study, the functional genes related to limonene bioconversion were investigated using genome and transcriptome sequences analysis. The results showed that the *P. digitatum* DSM 62840 genome was estimated to be 29.09 Mb and it encoded 9,086 protein-encoding genes. The most annotated genes were associated to some protein metabolism and energy metabolism functions. When the threshold for differentially expressed genes (DEGs) was set at twofold ratio, a total of 4,128, and 4,148 DEGs were identified in P_L_12h (limonene-treated condition) compared with P_0h (blank) and P_12h (limonene-untreated blank), respectively. Among them, the expression levels of genes involved in the biosynthesis of secondary metabolites, energy metabolism and ATP-binding cassette (ABC) transporters were significantly altered during the biotransformation. And the reliability of these results was further confirmed by quantitative real-time polymerase chain reaction (RT-qPCR). Moreover, we found that the enzyme participated in limonene biotransformation was inducible. This enzyme was located in the microsome, and it was inhibited by cytochrome P450 inhibitors. This indicated that the cytochrome P450 may be responsible for the limonene bioconversion. Several differentially expressed cytochrome P450 genes were further identified, such as PDIDSM_85260 and PDIDSM_67430, which were significantly up-regulated with limonene treatment. These genes may be responsible for converting limonene to α-terpineol. Totally, the genomic and transcriptomic data could provide valuable information in the discovery of related-genes which was involved in limonene biotransformation, pathogenicity of fungi, and investigation of metabolites and biological pathways of strain DSM 62840.

## Introduction

Limonene, a cyclic monoterpene, is the main volatile constituent of citrus fruits and other plant essential oils. It is usually discarded owing to its low aroma value and low chemical stability. While the oxyfunctionalized compounds of limonene have unique aroma and flavors with high economic value and pharmacological action (Sales et al., 2019). α-Terpineol is one of the bio-transformation products from limonene, and it has important applications in daily chemical, pharmaceutical, food, and flavor industries due to its pleasant odor which is similar to lilac (Molina et al., 2019). Moreover, it has a significant inhibitory effect on the proliferation of different cancer cell (Hassan et al., 2010), anti-oxidant and anti-proliferative action (Bicas et al., 2011; Khaleel et al., 2018), anti-microbial activity (Prakash et al., 2015), anti-inflammatory activity (Felipe et al., 2017; Held et al., 2007), anti-ulcer activity (Matsunaga et al., 2000), and presents good performance as an anti-convulsant agent (de Sousa et al., 2007). In addition, α-terpineol is an important intermediate in organic synthesis, and it could be used as a raw material for preparing dihydroterpineol, terpinyl acetate, geraniol, and so on. The conversion of limonene to α-terpineol is therefore economically attractive and will form a huge market. Recently, the price of α-terpineol is as high as US$ 1097.41/g, while it is only US$ 68.79/mL for the substrate limonene^1^.

The researches on the biotransformation of limonene have been reported for decades, including the isolation of microorganisms (Rottava et al., 2010a, b; Badee et al., 2011; Bier et al., 2017), the optimization of bio-transformation process (Bicas et al., 2008; Bicas et al., 2010; Molina et al., 2019) and the factors affecting the biotransformation (Prieto et al., 2011; Tai et al., 2016). Nevertheless, industrial applications of the limonene bioconversion are still limited because of the substrate inhibition in the liquid medium and the difficulty of the product isolation. These problems will result in the low yields of the end products. It is reported that the purified enzyme systems could not only solve these problems but also could confer a higher biotransformation rate and stereo-selectivity during bioconversions, and this will minimize the production of undesirable isomer and enantiomer (Gounaris, 2010). However, the purification of the enzymes related to the biotransformation has seldom been reported because of the complexity, time-consuming and high-cost of this process. Therefore, the molecular mechanism of limonene biotransformation is still unknown. To fill this gap, the limonene-converting mechanism was studied through incorporating genomics, transcriptomics, and bioinformatics.

*Penicillium digitatum* is a major fungal pathogen of citrus fruits, and it is reported that this fungal had the potential application in detoxifying monoterpenes (Molina et al., 2013). In our previous study, it is observed that *P. digitatum* strain DSM 62840 could convert limonene into α-terpineol with high regioselectivity (Tai et al., 2016). And the differentially expressed proteins at different periods of limonene biotransformation were explored using the isobaric tag for relative and absolute quantitation (iTRAQ) (Zhang et al., 2016). However, the detailed bioconversion pathways, regulation mechanisms and key enzymes remained unknown. Therefore, the genome and transcriptome of *P. digitatum* DSM 62840 have been collected to investigate the possible molecular mechanism during this biotransformation process. This work will provide a solid base for further investigation of the genetics of limonene bioconversion in strain DSM 62840.

## Materials and Methods

### Strain and Reagents

*Penicillium digitatum* DSM 62840 was purchased from the German Collection of Microorganisms and Cell Cultures (DSMZ, Braunschweig, Germany). Glucose, ethylene diamine tetraacetic acid (EDTA), 12 H_2_O⋅Na_2_HPO_4_, 2 H_2_O⋅NaH_2_PO_4_, and 1,10-phenanthroline were purchased from Sinopharm Chemical Reagent Co., Ltd. (Shanghai, China). *R*-(+)-Limonene and *R*-(+)-α-terpineol were obtained from Sigma Chemical Company (St. Louis, MO, United States). Hydrochloride (SKF-525A) and dithiothreitol (DTT) were purchased from Shanghai Aladdin Bio-Chem Technology Co., Ltd. (Shanghai, China). Malt extract was obtained from Beijing Shuangxuan Microbe Culture Medium Products Factory (Beijing, China). Yeast extract and peptone were purchased from Beijing Aoboxing Bio-Tech Co., Ltd. (Beijing, China). Phenylmethylsulfonyl fluoride (PMSF) was purchased from Shanghai Macklin Biochemical Co., Ltd. (Shanghai, China).

### Genomic DNA Isolation and Sequencing

Strain DSM 62840 was cultivated and stored on potato dextrose agar (PDA) (Qingdao Hope Bio-Technology Co., Ltd., Shandong, China). And then the 1-week-old culture dishes incubated at 24°C were flooded with sterile distilled water containing 0.1% Tween 80. The concentration of spore suspension was adjusted to 1.5 × 10^7^ spores/mL. Subsequently, 1 mL of spore suspensions were aseptically transferred into 250 mL Erlenmeyer flasks containing 100 mL of MYB medium (yeast extract = 30 g/L, malt extract = 20 g/L, peptone = 10 g/L, and glucose = 10 g/L), and then it was incubated at 24°C with 150 rpm for 2 days (Zhang et al., 2016). Then, the fungal mycelia were harvested by vacuum filtration, and stored at −80°C after quick freezing in liquid nitrogen. Genomic DNA was extracted from the fungal mycelia by using Ezup column fungi genomic DNA purification kit (Sangon Biotech Co., Ltd., Shanghai, China). The genome sequencing was performed using high-throughput Illumina HiSeq X Ten sequencing and the PacBio RSII sequencing platform at Beijing Genomics Institute (BGI, Shenzhen, China).

### Genome Assembly and Gene Annotation

Firstly, the reads of low complexity, low quality and sequencing errors were filtered from the raw data to obtain clean data. The following operations were performed in order to filter the raw data of the Illumina HiSeq X Ten system: (1) reads with a certain proportion of low quality (20%) bases were removed, (2) reads with a certain proportion of Ns’ base (10%) were removed, and (3) adapter and duplication contaminations were removed. The following operations were performed in order to filter the raw data of PacBio RSII platform: (1) polymerase reads with length < 1,000 bp were filtered out, (2) polymerase reads with quality score < 0.8 were filtered out, (3) adapter sequences were filtered out and then subreads were produced, (4) subreads with length < 1,000 bp were filtered out, and (5) subreads with quality score < 0.8 were filtered out for further analysis.

Secondly, subreads were corrected using the Proovread 2.12 to produce credible Corrected Reads. The parameters of Proovread were: -t 4 –coverage 60 –mode sr. Corrected Reads were assembled using the Celera Assembler 8.3 and Falcon v0.3.0. The parameters of Celera Assembler were: doTrim_initialQualityBased = 1, doTrim_finalEvidenceBased = 1, doRemoveSpurReads = 1, doRemoveChimericReads = 1, and -d properties -U. The parameters of Falcon were: -v -dal8 -t32 -h60 -e.96 -l500 -s100 -H3000. And then GATK v1.6-13 was used to correct single bases from the assembly results. The parameters of GATK were: -cluster 2 -window 5 -stand_call_conf 50 -stand_emit_conf 10.0 -dcov 200 MQ0 ≥ 4. After that, SSPACE_Basic_v2.0 was used to construct the scaffold. And then pbjelly2 15.8.24 was used to fill gaps (default parameters).

Thirdly, genes were predicted by using genewise 2.20, SNAP v 2010-07-28, Augustus 3.2.1 and GeneMarkes 4.21. rRNA, tRNA, and sRNA sequences were identified by RNAmmer (version 1.2), tRNAscan-SE software (version 1.3.1) and Rfam (version 9.1), respectively. Transposon sequence analysis was implemented for the assembled gene sequences with the transposon Repbase database, RepeatProteinMasker software and *De novo*. Tandem repeat sequences were searched in the DNA sequence with Tandem Repeat Finder (TRF version 4.04).

Finally, the protein coding genes were annotated with several complementary approaches. The genes were aligned with diverse protein databases, including Gene Ontology (GO) (version 2016-01-12), Kyoto Encyclopedia of Genes and Genomes (KEGG) (version 76), Cluster of Orthologous Groups of proteins (COG) (version 2014-11-10), Swiss-Prot (version 2016-01), Trembl (version 2016-01), Non-redundant (NR) (version 2015-05-31), EggNOG (version 4.5), Pathogen Host Interactions (PHI) (version 4.0), Carbohydrate-active enzymes (CAZymes) (version 2016-04), and fungal Cytochrome P450 Database (version 1.1). Moreover, AntiSMASH 5.0 was employed to predict the gene clusters of secondary metabolites. The parameter settings were maintained as the default parameter values.

### RNA Isolation, Integrity Examination, and RNA-Seq Library Preparation

Three samples at different periods during limonene biotransformation were selected for RNA sequencing, and they were blank (P_0h), limonene-treated condition (P_L_12h), and limonene-untreated blank (P_12h). Two biological replicates for each treatment were used for RNA isolation, integrity examination and library preparation. In brief, the strain DSM 62840 was cultivated in MYB medium for 48 h with 150 rpm at 24°C as described previously, and the fungal mycelia were harvested and the total RNA was extracted using the Trizol method (Ali et al., 2017). This sample was labeled as the blank (P_0h). At the same time, the RNA from fungal mycelia in the sample of limonene-treated condition (P_L_12h) was extracted after a 12 h-incubation period with 840 mg/L limonene. And the RNA from the sample of limonene-untreated blank (P_12h) was obtained after a 12 h-incubation period without limonene. Agilent 2100 Bioanalyzer (Agilent RNA 6000 Nano Kit) was used for the determination of the RNA integrity.

Subsequently, mRNA was enriched by using oligo (dT) magnetic beads. Fragmentation was carried out using fragmentation buffer under elevated temperature. The cleaved RNA fragments were then transcribed into first-strand cDNA using reverse transcriptase and random hexamer primers. The second strand cDNA synthesis was subsequently performed using DNA Polymerase I and RNase H. Double stranded cDNA was processed with end-repair and adapter-ligation. Adaptor ligated fragments were selected according to the size. PCR was accomplished to selectively enrich and amplify the fragments. Finally, after validating on an Agilent 2100 Bioanalyzer and ABI StepOnePlus Real-Time PCR System, the cDNA library was sequenced using HiSeq X Ten.

### Analysis of RNA-Seq Data

The raw sequences were preprocessed by removing low-quality reads and the reads containing adapters or ploy-N using SOAPnuke v1.5.2^2^ to get clean reads. The clean reads were aligned to the reference genome of strain DSM 62840 using HISAT v2.0.4. The expression level of gene was estimated using the Fragments Per Kilobase per Million mapped fragments (FPKM). The differential expression analysis was performed using the DESeq2 (Love et al., 2014). Genes with an adjusted *P*-value ≤ 0.05 and a fold change ≥ 2.0 were considered to be differentially expressed. GO term and KEGG pathway enrichment analysis of differentially expressed genes (DEGs) were further performed with phyper which is a function of R. A false discovery rates (FDRs) ≤ 0.01 were considered to be significantly enriched.

### Effect of Inhibitors on the Enzymatic Activity

*Penicillium digitatum* DSM 62840 was cultivated in MYB medium for 2 days with 150 rpm at 24°C as described previously. The fungal mycelium was harvested by vacuum filtration, and was washed twice using buffer 1 (50 mM phosphate buffer, pH 7.0 containing 1 mM DTT, 1 mM PMSF, and 1 mM EDTA). After washing twice, the pellet (20 g wet weight) was resuspended in 200 mL buffer 1. The pellets were disrupted for 5 times by high pressure cell disrupter (Guangzhou Juneng Nano & Bio Technology Co., Ltd., Guangzhou, China) with 1, 500 bar. Then the homogenate was centrifuged at 10,000 rpm for 20 min. The supernatant was further clarified by re-centrifuging under the same conditions and labeled as post-mitochondrial fractions (F1). The obtained F1 (1 mL) was incubated with 1 mM inhibitor (cytochrome P450 inhibitor SKF-525A and metalloproteinase inhibitor 1,10-phenanthroline) and 840 mg/L limonene at 24°C and 150 rpm for 12 h. The biotransformation activity was monitored after 12 h by solid-phase microextraction/gas chromatography-mass spectrometry (SPME/GC-MS) (Agilent Technologies, Palo Alto, CA, United States) according to the method of Zhang et al. (2019). The control without inhibitor was also carried out. The experiments were performed in triplicate. One unit of enzyme activity was defined as producing 1 mg/L of α-terpineol per hour. The content of α-terpineol was quantitative analyzed by external standard method. The standard curve was *y* = 131423*x* + 2 × 10^6^, *R*^2^ = 0.999 [*x* is the concentration of α-terpineol (mg/L), and y is the peak area].

### Isolation of Microsomes and Determination of the Enzymatic Activity

Microsomes were obtained by centrifuging the F1 at 40,000 rpm for 90 min using an ultracentrifuge (Beckman Coulter, Inc., United States). The obtained supernatant was labeled as cytosol fractions (F2). The obtained precipitate was divided into two parts. One part was dissolved with buffer 2 (20 mM phosphate buffer, pH 7.0 containing 1 mM DTT, 1 mM PMSF, and 1 mM EDTA) and was centrifuged at 12,000 rpm for 40 min. The obtained supernatant was labeled as microsomal fractions (F3). The other part was dissolved in buffer 2 with 1% Triton X100 and it was centrifuged. Clear yellow supernatant was taken and labeled as F4.

Totally 1 mL of protein solution (F1, F2, F3, and F4) was incubated with 840 mg/L of limonene. The incubation was carried out in a 2 mL centrifuge tube at 24°C and 150 rpm for 12 h. Then the sample was analyzed using SPME/GC-MS as described previously. The experiments were performed in triplicate. Besides, the protein content of F1, F2, F3, and F4 solution were determined by BCA kit (Beijing Dingguo Changsheng Biotechnology Co. Ltd., Beijing, China).

### Inducibility of the Enzyme

The strain of DSM 62840 was cultivated in MYB medium for 48 h with shaking (150 rpm) at 24°C, and the fungal mycelia were harvested as the blank (P_0h). At the same time, fungal mycelia in limonene-treated condition (P_L_12h) were obtained after a 12 h-incubation period with 840 mg/L limonene. And fungal mycelia in limonene-untreated blank (P_12h) were harvested after a 12 h-incubation period without limonene. The F4 solution obtained from the three samples were performed as described previously. Totally 1 mL of F4 solution was incubated with 840 mg/L limonene. Incubation was carried out in a 2 mL centrifuge tube at 24°C and 150 rpm for 12 h. Then the sample was analyzed using SPME/GC-MS as described previously. The experiments were performed in triplicate.

### Quantitative Real-Time Polymerase Chain Reaction (RT-qPCR)

Ten genes (PDIDSM_55100, PDIDSM_01820, PDIDSM_85260, PDIDSM_53370, PDIDSM_47510, PDIDSM_12860, PDIDSM_67430, PDIDSM_08220, PDIDSM_577403, and PDIDSM_86880) associated with the synthesis of secondary metabolites, energy metabolism and cytochrome P450 (CYPs) were chosen for the confirmation of the transcriptome data by RT-qPCR. The RNA in P_0h, P_12h, and P_L_12h were extracted as described previously. cDNA was acquired by Evo M-MLV RT kit (Accurate Biotechnology Co., Ltd., Wuhan, China). RT-qPCR analysis was performed on qTOWER 2.2 (Analytik Jena AG, Germany) using the SYBR Green Premix Pro Taq HS qPCR Kit (Accurate Biotechnology Co., Ltd., Wuhan, China) under the following conditions: 95°C for 30 s, followed by 40 cycles 95°C for 5 s, 60°C for 30 s. All reactions were performed in a total sample volume of 10 μL (5.0 μL of 2 × SYBR Green Pro Taq HS Premix, 1.0 μL of primers, 0.5 μL of cDNA, 3.5 μL of ddH_2_O). Primers were designed using Primer-BLAST (Supplementary Table S1). β-Actin was used as an internal control. RT-qPCR experiment was repeated three times, each sample having three technique replicates. The relative expression level of the genes was calculated using the 2^–ΔΔCT^ method and it was reported as mRNA expression relative to P_0h.

### Statistical Analysis

Data was expressed as mean ± standard error of the mean (SEM). Statistical analysis was determined using the Mann–Whitney test (GraphPad Software, Inc., San Diego, CA, United States). The level of significance was established at *p* < 0.05 and *p* < 0.01.

## Results and Discussion

### Evaluation of the Genome Data

The results showed that 4,629 Mb HiSeq-data and 3,757 Mb PacBio-data were generated in *P. digitatum* DSM 62840. The assembly process produced 31 scaffolds with an estimated genome size of 29.09 Mb and an N50 of 6,602,426 bp. A total of 9,086 protein-encoding genes were predicted in strain DSM 62840 genome by publicly available databases. The length of exons and intron areas were 11,895,138 and 1,017,612 bp, respectively (Table 1). With regard to RNA, totally 211 tRNAs, 43 rRNAs, 23 sRNAs, 14 snRNAs, and 81 miRNAs were predicted. Currently, four genomes of *P. digitatum* species (*P. digitatum* Pd1, *P. digitatum* PHI26, *P. digitatum* Pd01-ZJU, and *P. digitatum* PDC 102) were available in the National Center for Biotechnology Information (NCBI) database^3^ (Sun et al., 2011, 2013; Marcet-Houben et al., 2012; Julca et al., 2016). Assembly statistics of the different *P. digitatum* genomes were summarized in Supplementary Table S2, showing similar GC content but significantly different genome sizes. Additionally, the number of the scaffolds of *P. digitatum* DSM 62840 was less than that of strain Pd1, PHI26, and Pd01-ZJU.

**TABLE 1:** Genome assembly and annotation statistics of *Penicillium digitatum* DSM 62840.

**Assembly statistics**	
Scaffold total number	31
Scaffold total length (bp)	26,577,035
Scaffold N50 length (bp)	6,602,426
Scaffold N90 length (bp)	959,294
Scaffold Gap number (bp)	103,949
GC content (%)	48.87
**Gene statistics**	
Gene assembly (Mb)	29.09
Number of coding sequence genes	9,086
Number of exons	24,363
Number of introns	15,048
Average length of coding sequence genes (bp)	1,276.99
Average exons length (bp)	488.25
Average introns length (bp)	67.72
**Functional annotations**	
CAZy	197
COG	997
KEGG	3,706
KOG	1,888
NOG	7,480
NR	9,245
P450	703
PHI	804
SwissProt	2,681
TrEMBL	9,017

**FIGURE 1:**
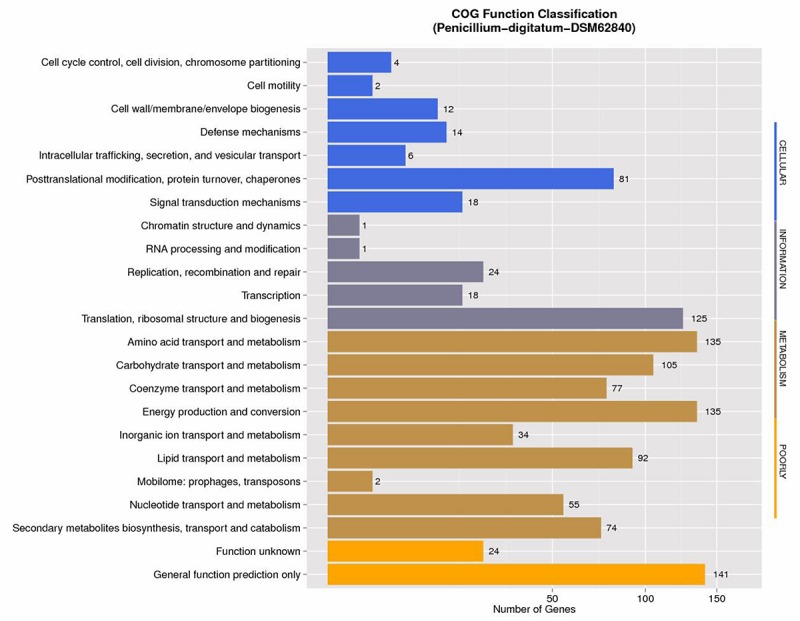
COG function classification of *Penicillium digitatum* DSM 62840 genome.

The annotations with NCBI NR, GO, COG, NOG, KEGG, CAZy, SwissProt, PHI, P450, and TrEMBL protein databases were shown in Supplementary Table S3. NCBI COG mapping showed that 997 proteins were assigned to COG categories (Figure 1). The most gene-rich COG classifications were “general functional prediction only,” “energy production and conversion,” “amino acid transport and metabolism,” “translation, ribosomal structure and biogenesis,” “carbohydrate transport and metabolism,” “lipid transport and metabolism,” and “posttranslational modification, protein turnover, and chaperones.” These findings were suggestive of the presence of a variety of protein metabolism and energy metabolism functions that could enable better absorption and transformation of nutrients from the substrates. In order to further understand the gene functions in strain DSM 62840, 7,480, 1,888, and 3,706 genes were assigned to the NOG, KOG, and KEGG database, respectively (Supplementary Figure S1). It is similar to the COG annotation that some metabolism and biosynthesis categories were highly enriched.

### Evaluation of the Transcriptome Data

*Penicillium digitatum* DSM 62840 has been proved to be an efficient biocatalyst to transform limonene to α-terpineol when the conversion time was 12 h. Therefore the samples from three different periods were selected to perform RNA-seq in order to find potential candidate genes involved in limonene biotransformation. After removal of low quality reads, the number of clean reads ranged from 42.04 to 43.02 million. And approximately 75.32–80.51% of the clean reads were mapped to the *P. digitatum* DSM 62840 reference genome, indicating that the quality of transcriptome sequencing data was reliable (Supplementary Table S4).

Significant changes in the transcriptome profiles were observed at various periods during limonene biotransformation. We used an adjusted *P*-value ≤ 0.05 and a fold change ≥ 2.0 as thresholds to identify the DEGs. Totally 448, 4,128, and 4,148 DEGs were identified in these three comparisons (“P_0h vs. P_12h”, “P_0h vs. P_L_12h,” and “P_12h vs. P_L_12h”), respectively (Table 2). The number of up-regulated DEGs was slightly larger than that of down-regulated DEGs. A total of 260/188, 2,080/2,048, and 2,073/2,075 up-/down-regulated DEGs were identified in these three comparisons, respectively. Apart from this, the number of DEGs in “P_0h vs. P_12h” was less than other two comparisons, owing to no limonene induction. And the number of DEGs or up-/down-regulated DEGs in “P_0h vs. P_L_12h” was almost the same with that in “P_12h vs. P_L_12h.”

**TABLE 2:** The number of differentially expressed genes (≥2-fold change) at various periods during limonene biotransformation.

**Group**	**Gene number**		
	**Total**	**Up-regulated**	**Down-regulated**
P_0h vs. P_12h	448	260	188
P_0h vs. P_L_12h	4,128	2,080	2,048
P_12h vs. P_L_12h	4,148	2,073	2,075

**FIGURE 2:**
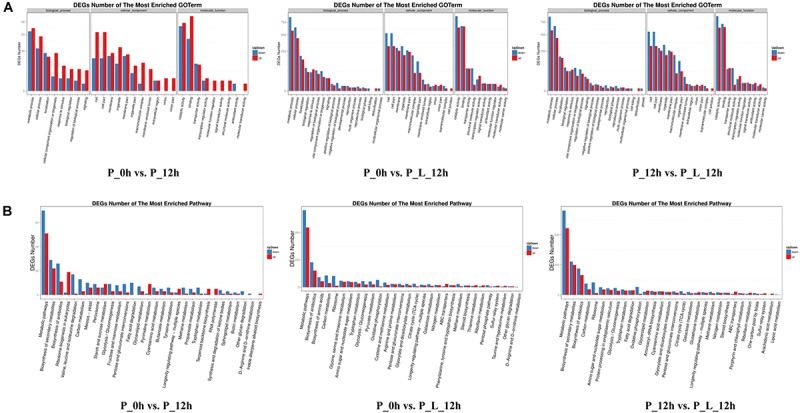
Functional analysis of differentially expressed genes (≥2-fold change) at various periods during limonene biotransformation. **(A)** Gene Ontology (GO) functional annotation and classification of differentially expressed genes, **(B)** KEGG functional annotation of differentially expressed genes. P_0h, blank; P_12h, limonene-untreated blank; P_L_12h, limonene-treated condition.

In order to identify the biological processes, molecular functions and cellular components contributing to limonene biotransformation, GO term analysis were executed on up-/down-regulated DEGs (Figure 2A). The predominant GO categories included cell, cell parts, membrane and organelles in cellular components, and catalytic activity, binding, transporter activity and structural molecule activity in molecular function. In addition, the main biological process categories were associated with metabolic process, cellular process, localization and biological regulation in both “P_0h vs. P_L_12h” and “P_12h vs. P_L_12h.” These findings were consistent with the results of previous iTRAQ data (Zhang et al., 2016). Moreover, a KEGG analysis was accomplished to find the active pathways in limonene biotransformation. The up-/down-regulated DEGs were mainly annotated to the following categories: metabolic pathways, biosynthesis of antibiotics, biosynthesis of amino acids, carbon metabolism, ribosome, biosynthesis of secondary metabolites, which exemplified that limonene activated the specific metabolic process of cells (Figure 2B). Notably, most of the genes involved in tricarboxylic acid cycle (TCA cycle) and oxidative phosphorylation terms were down-regulated during the bioconversion, and the genes involved in ATP-binding cassette (ABC) transporters terms were up-regulated.

### Identification of Secondary Metabolite Biosynthetic Genes Involved in Limonene Biotransformation

The biosynthetic genes of secondary metabolites are often in contiguous gene clusters. A number of biosynthetic genes responsible for the synthesis of secondary metabolites were forecasted through antiSMASH 5.0, such as terpene, non-ribosomal peptide synthetases (NRPS), and polyketide synthases (PKS). A total of 32 gene clusters were distinguished in the strain DSM 62840 genome, including 4 Nrps-like, 7 T1pks, 5 terpenes, 7 Nrps, 1 beta-lactone, 1 T1pks-terpene, 1 T1pks-Nrps-like, 1 T3pks-Nrps, 3 T1pks-Nrps, 1 indole-T1pks, and 1 terpene-Nrps-like. And 26 PKS and NRPS gene clusters were revealed, accounting for 81.25% of the total number of predicted gene clusters. The details of the gene clusters were displayed in Supplementary Table S5. Similar results were also gained in another two *Penicillium* sp., *P. digitatum* PHI26 and *Penicillium chrysogenum*, which had 13 and 24 NRPSs, 14 and 23 PKS, 3 and 2 NRPS-PKS hybrids, and one and zero prenyltransferase (DMATSs) gene clusters, respectively (Marcet-Houben et al., 2012).

Terpene is one of the most important secondary metabolites in fungi, and it has been widely applied in food, pharmaceutical and cosmetics fields (Yu et al., 2019). Terpene is built from the simple five carbon precursor molecules dimethylallyl diphosphate (DMAPP) and isopentenyl diphosphate (IPP), and these precursors are produced from acetyl-CoA via the mevalonate (MVA) pathway. Therefore, we searched the strain DSM 62840 genome in the “terpenoid backbone biosynthesis” pathway and identified 17 key enzymes distributed in the MVA pathway (Supplementary Table S6), but not the 2-C-methyl-D-erythritol 4-phosphate/1-deoxy-Dxylulose 5-phosphate (MEP/DOXP) pathway (Supplementary Figure S2). This suggested that *P. digitatum* DSM 62840 can proceed through the MVA pathway instead of MEP/DOXP pathway like most fungi. The transcriptional changes in response to limonene were observed in a set of genes associated with terpene biosynthesis (Table 3). The expression levels of PDIDSM_00870 encoding hexaprenyl-diphosphate synthase (hexPS, COQ1), PDIDSM_339802 encoding prenyl protein peptidase (RCE1 and FACE2), PDIDSM_75960 encoding prenylcysteine oxidase/farnesylcysteine lyase (FCLY), PDIDSM_12890 encoding diphosphomevalonate decarboxylase (MVD and mvaD), PDIDSM_12330 encoding STE24 endopeptidase (STE24), PDIDSM_88970 encoding acetyl-CoA *C*-acetyltransferase (atoB) and PDIDSM_69340 encoding hydroxymethylglutaryl-CoA reductase (HMGCR) had a lower expression in P_L_12h than that in P_0h and P_12h, respectively. Whereas the expression levels of PDIDSM_04030 encoding terpenoid synthase in P_L_12h was higher than that in P_0h and P_12h. Terpenoid synthase could catalyze the IPP and its isomer DMAPP to produce geranyl pyrophosphate (GPP), farnesyl diphosphate (FPP), and geranylgeranyl pyrophosphate (GGPP). And these products were the precursors of monoterpenes, sesquiterpenes, and diterpenes, respectively. This suggested that limonene could stimulate the expression of these genes in strain DSM 62840.

**TABLE 3:** Identification of differentially expressed genes (≥2-fold change) involved in limonene biotransformation by RNA sequencing.

**Gene ID**	**Protein description**	**Functional annotation**	**log2 FoldChange (P_0h vs. P_12h)**	***P*-value**	**log2FoldChange (P_0h vs. P_L_12h)**	***P*-value**	**log2FoldChange (P_12h vs. P_L_12h)**	***P*-value**
**The secondary metabolite biosynthetic genes involved in limonene biotransformation**								
PDIDSM_88970	Acetyl-CoA *C*-acetyltransferase	Acetyl-CoA *C*-acetyltransferase [EC:2.3.1.9]	0.52	0.03	−1.67	6.18E-08	−2.23	8.71E-15
PDIDSM_69340	Hydroxymethylglutaryl-CoA reductase (NADPH)	Hydroxymethylglutaryl-CoA reductase (NADPH) [EC:1.1.1.34]; sterol-4alpha-carboxylate 3-dehydrogenase (decarboxylating) [EC:1.1.1.170]	−0.91	0.001	−2.16	1.92E-12	−1.20	7.87E-05
PDIDSM_12890	Diphosphomevalonate decarboxylase	Diphosphomevalonate decarboxylase [EC:4.1.1.33]	−0.23	0.35	−2.09	6.43E-07	−1.87	8.53E-06
PDIDSM_75960	Prenylcysteine oxidase/farnesylcysteine lyase	Prenylcysteine oxidase/farnesylcysteine lyase [EC:1.8.3.5 1.8.3.6]	−0.04	0.87	−2.51	5.46E-08	−2.48	2.47E-08
PDIDSM_12330	STE24 endopeptidase	STE24 endopeptidase [EC:3.4.24.84]	−0.01	0.97	−2.19	6.82E-09	−2.20	6.58E-13
PDIDSM_16240	Protein farnesyltransferase/geranylgeranyltransferase type-1 subunit alpha	Choline-sulfatase [EC:3.1.6.6]; protein farnesyltransferase/geranylgeranyltransferase type-1 subunit alpha [EC:2.5.1.58 2.5.1.59] L-serine/L-threonine ammonia-lyase [EC:4.3.1.17 4.3.1.19]	0.32	0.17	1.92	2.80E-07	1.58	8.21E-06
PDIDSM_04030	Terpenoid synthase	Ophiobolin F synthase [EC:4.2.3.145]; geranylgeranyl diphosphate synthase, type III [EC:2.5.1.1 2.5.1.10 2.5.1.29]	−0.55	0.03	3.71	9.66E-36	4.29	1.42E-60
PDIDSM_00870	Hexaprenyl-diphosphate synthase	Hexaprenyl-diphosphate synthase [EC:2.5.1.82 2.5.1.83]	−0.60	0.12	−2.69	3.37E-11	−1.98	4.69E-04
PDIDSM_339802	Prenyl protein peptidase	Glycerol-3-phosphate *O*-acyltransferase/dihydroxyacetone phosphate acyltransferase [EC:2.3.1.15 2.3.1.42]; leucyl-tRNA synthetase [EC:6.1.1.4]; prenyl protein peptidase [EC:3.4.22.-]	−0.32	0.37	−3.05	2.91E-09	−3.05	2.91E-09
PDIDSM_55550	Mevalonate kinase	Tubulin alpha; mevalonate kinase [EC:2.7.1.36]	0.49	0.11	1.91	6.25E-06	1.37	8.89E-04
PDIDSM_55100	Hypothetical protein PDIP_55100	Farnesyl-diphosphate farnesyltransferase [EC:2.5.1.21]	−0.02	0.94	−2.72	1.32E-04	−2.71	7.94E-05
PDIDSM_86810	Hypothetical protein PDIP_86810	Aristolochene synthase [EC:4.2.3.9]	−0.02	0.97	−1.79	0.06	−1.77	0.06
PDIDSM_02860	Trichodiene synthase	Trichodiene synthase [EC:4.2.3.6]	0.41	0.39	3.76	0.004	2.15	0.08
PDIDSM_01820	14-alpha sterol demethylase	Sterol 14-demethylase [EC:1.14.13.70]	0.74	0.03	−2.90	1.80E-21	−3.69	1.15E-21
PDIDSM_52510	Squalene epoxidase-like protein	squalene monooxygenase [EC:1.14.14.17]	1.37	0.002	−3.03	4.13E-07	−4.65	2.49E-13
**The genes involved in energy metabolism**								
PDIDSM_47510	Malate dehydrogenase	Malate dehydrogenase [EC:1.1.1.37]	0.04	0.83	−2.15	9.48E-09	−2.20	8.84E-10
PDIDSM_28150	Isocitrate dehydrogenase, putative	Isocitrate dehydrogenase (NAD+) [EC:1.1.1.41]	0.09	0.72	−1.98	9.80E-08	−2.08	5.45E-08
PDIDSM_43060	Alpha-ketoglutarate dehydrogenase complex subunit Kgd1, putative	2-oxoglutarate dehydrogenase E1 component [EC:1.2.4.2]	0.35	0.16	−1.88	1.16E-09	−2.26	6.88E-15
PDIDSM_53370	Succinate dehydrogenase cytochrome b560 subunit	Succinate dehydrogenase (ubiquinone) cytochrome b560 subunit	−0.32	0.14	−1.74	2.19E-12	−1.42	2.01E-08
PDIDSM_04620	Citrate synthase	Citrate synthase [EC:2.3.3.1]	–	–	6.00	2.47E-05	5.81	2.81E-05
PDIDSM_36580	Phosphoenolpyruvate carboxykinase AcuF	Phosphoenolpyruvate carboxykinase (ATP) [EC:4.1.1.49]	0.42	0.16	−3.37	8.25E-26	−3.84	1.49E-45
PDIDSM_67880	Pyruvate dehydrogenase E1 beta subunit PdbA, putative	Pyruvate dehydrogenase E1 component beta subunit [EC:1.2.4.1]	−0.03	0.90	−2.04	3.81E-14	−2.01	6.72E-12
PDIDSM_16200	Pyruvate carboxylase, putative	Pyruvate carboxylase [EC:6.4.1.1]	0.17	0.49	−1.56	9.77E-08	−1.74	1.96E-08
PDIDSM_45360	Mitochondrial aconitate hydratase, putative	Aconitate hydratase [EC:4.2.1.3]	0.64	0.04	−2.15	3.69E-08	−2.87	2.01E-19
PDIDSM_01580	Dihydrolipoamide succinyltransferase, putative	2-oxoglutarate dehydrogenase E2 component (dihydrolipoamide succinyltransferase) [EC:2.3.1.61]	−0.06	0.79	−2.42	1.84E-14	−2.37	5.26E-16
PDIDSM_12860	Cytochrome c oxidase subunit VIIc	Cytochrome c oxidase subunit 7c	0.04	0.85	−3.03	2.17E-26	−3.09	8.56E-25
PDIDSM_34810	Ubiquinol-cytochrome C reductase complex core protein 2, putative	Ubiquinol-cytochrome c reductase core subunit 2	0.01	0.98	−2.21	4.71E-15	−2.22	5.99E-14
**The genes involved in ABC transports**								
PDIDSM_49740	ABC bile acid transporter, putative	ATP-binding cassette, subfamily C (CFTR/MRP), member 1;ATP-binding cassette, subfamily B (MDR/TAP), member 1	0.6	0.01	2.75	1.49E-21	2.11	4.01E-15
PDIDSM_06070	ABC multidrug transporter, putative	ATP-binding cassette, subfamily C (CFTR/MRP), member 1; ATP-binding cassette, subfamily B (MDR/TAP), member 1	0.52	0.17	1.76	9.23E-04	1.16	0.01
PDIDSM_72790	ABC a-pheromone efflux pump	ATP-binding cassette, subfamily B (MDR/TAP), member 1	0.04	0.89	1.18	1.43E-04	1.13	1.58E-4
PDIDSM_719402	Hypothetical protein PDIG_12480	ATP-binding cassette, subfamily B (MDR/TAP), member 6	−0.14	0.58	2.71	2.25E-11	2.85	7.28E-13
PDIDSM_20430	Hypothetical protein PDIG_51230	ATP-binding cassette, subfamily D (ALD), peroxisomal long-chain fatty acid import protein	0.09	0.77	1.41	0.002	1.29	0.003
PDIDSM_667602	ABC transporter, putative	ATP-binding cassette, subfamily G (WHITE), member 2, PDR	0.38	0.15	7.78	1.12E-217	7.37	1.69E-194
PDIDSM_17720	Hypothetical protein PDIG_45780	ATP-binding cassette, subfamily G (WHITE), member 2, PDR	0.65	0.23	5.89	6.06E-30	4.87	1.49E-18
PDIDSM_66760	ABC transporter, putative	ATP-binding cassette, subfamily G (WHITE), member 2, PDR	0.28	0.19	2.51	3.99E-23	2.20	2.15E-18
PDIDSM_667603	ABC multidrug transporter, putative	ATP-binding cassette, subfamily G (WHITE), member 2, PDR	0.05	0.92	2.64	5.54E-04	2.51	0.002
PDIDSM_667608	ABC multidrug transporter, putative	ATP-binding cassette, subfamily G (WHITE), member 2, SNQ2	−0.32	0.39	3.72	4.33E-18	4.11	1.81E-56
PDIDSM_667609	ABC transporter, putative	ATP-binding cassette, subfamily G (WHITE), member 2, SNQ2	−0.21	0.71	3.26	2.92E-08	3.52	1.31E-05
PDIDSM_17650	ABC drug exporter AtrF	ATP-binding cassette, subfamily G (WHITE), member 2, SNQ2	0.25	0.63	0.39	0.003	2.65	0.01
PDIDSM_76180	ABC efflux transporter, putative	ATP-binding cassette, subfamily G (WHITE), member 2, SNQ2	0.42	0.29	2.89	5.03E-12	2.40	1.52E-11
PDIDSM_78490	ABC transporter, putative	ATP-binding cassette, subfamily G (WHITE), member 2, SNQ2	0.69	0.08	2.05	6.35E-05	1.25	0.006
**Potential CYPs involved in limonene biotransformation**								
PDIDSM_85260	Hypothetical protein PDIG_38620	Transcriptional regulatory protein GAL4	–	–	11.83	1.32E-28	11.68	9.11E-30
PDIDSM_67430	Bifunctional P450:NADPH-P450 reductase	Cytochrome P450/NADPH-cytochrome P450 reductase [EC:1.14.14.1 1.6.2.4]	–	–	7.05	1.19E-07	6.88	1.09E-07
PDIDSM_86880	Hypothetical protein PDIP_86880	3-hydroxyphenylacetate 6-hydroxylase [EC:1.14.13.63]; unspecific monooxygenase [EC:1.14.14.1]; methylglutaconyl-CoA hydratase [EC:4.2.1.18]; averantin hydroxylase [EC:1.14.13.174]; benzoate 4-monooxygenase [EC:1.14.13.12]	0.58	0.06	3.31	6.90E-13	2.68	1.28E-09
PDIDSM_67660	Cytochrome P450 alkane hydroxylase, putative	Cytochrome P450 monooxygenase; unspecific monooxygenase [EC:1.14.14.1]	0.39	0.28	−3.27	6.97E-08	−3.69	1.55E-09
PDIDSM_67750	Cytochrome P450 monooxygenase, putative	Para-aminobenzoate synthetase [EC:2.6.1.85]; cytochrome P450/NADPH-cytochrome P450 reductase [EC:1.14.14.1 1.6.2.4]	−0.42	0.09	−3.56	5.72E-14	−3.11	9.07E-10
PDIDSM_08220	P450 family fatty acid hydroxylase, putative	cypD_E, CYP102A2_3 cytochrome P450/NADPH-cytochrome P450 reductase	0.59	0.005	−1.74	6.30E-08	−2.35	2.74E-14
PDIDSM_577403	Benzoate 4-monooxygenase cytochrome P450	Tryprostatin B 6-hydroxylase [EC:1.14.13.176]; ATP-binding cassette, subfamily B (MDR/TAP), member 1 [EC:3.6.3.44]; benzoate 4-monooxygenase [EC:1.14.13.12]; paired amphipathic helix protein Sin3a	−0.50	0.38	2.95	1.57E-04	3.83	4.87E-06
PDIDSM_42610	Hypothetical protein PDIG_49750	Benzoate 4-monooxygenase [EC:1.14.13.12]	0.002	0.99	3.65	0.02	3.55	0.02
PDIDSM_62110	Cytochrome P450 monooxygenase, putative	Tryprostatin B 6-hydroxylase [EC:1.14.13.176]; fatty acid synthase subunit beta, fungi type [EC:2.3.1.86]; benzoate 4-monooxygenase [EC:1.14.13.12]	−0.29	0.35	3.02	1.91E-14	3.33	2.29E-25
PDIDSM_12990	Cytochrome P450 monooxygenase, putative	Benzoate 4-monooxygenase [EC:1.14.13.12]	−0.09	0.83	2.75	0.02	2.96	0.02
PDIDSM_0468015	Cytochrome P450, putative	Tryprostatin B 6-hydroxylase [EC:1.14.13.176]; benzoate 4-monooxygenase [EC:1.14.13.12]	−0.15	0.77	1.39	0.01	1.54	0.02

*“–” means that the gene was not detected.*

On the other hand, we found that there were 1 terpene-T1PKS (cluster 3), 1 terpene-NRPS-like (cluster 24) and 5 terpene gene clusters (the clusters of 9, 17, 19, 27, and 30) in the strain DSM 62840 genome. And cluster 3 and cluster 17 showed a 50 and 100% similarity to the PR-toxin biosynthetic gene cluster and deoxysambucinol/sambucinol/roridin E biosynthetic gene cluster, respectively. The clusters 3 and 17 were mainly responsible for the sesquiterpenoid biosynthetic process. Sesquiterpene synthases catalyze the cyclization of the acyclic precursor FPP to form monocyclic, bicyclic, and tricyclic sesquiterpenes. PDIDSM_86810 from cluster 3 was predicted to have the aristolochene synthase (ARI1) activity. ARI1 is a fungal sesquiterpene cyclase that could convert FPP to aristolochene. It is considered to be the precursor of highly oxygenated mycotoxins such us PR-toxin (Faraldos et al., 2016). Similarly, PDIDSM_02860 from cluster 17 annotated as trichodiene synthase (TRI5) was a sesquiterpene synthase that catalyzed the cyclization of FPP to form the bicyclic sesquiterpene hydrocarbon trichodiene and the coproduct pyrophosphate (PPi) (Vedula et al., 2007). Besides, cluster 30 probably took part in the biosynthesis of sesquiterpenoid and triterpenes. PDIDSM_55100 from cluster 30 was predicted to possess farnesyl-diphosphate farnesyltransferase (FDFT1) activity. FDFT1 catalyzes a two-step reaction in which two molecules of FPP are converted into one molecule of squalene. It is the first committed precursor of sterols and most other triterpenes (Kempinski et al., 2019). Its epoxidation to produce 2,3-oxidosqualene was catalyzed by PDIDSM_52510 encoding squalene epoxidase (SQLE), and the obtained product was then cyclized into lanosterol. PDIDSM_01820 was annotated as a sterol 14-demethylase (CYP51), and it catalyzed the removal of the 14 α-methyl group from lanosterol. This reaction is a necessary step in sterol biosynthesis. Several genes which were involved in the sterol-biosynthetic pathway were regulated by oxygen and heme levels, such as FDFT1, CYP51, SQLE, and HMGCR. And their expression was influenced by various transcription factors (Geisler et al., 2013). The results indicated that the transcription of gene FDFT1, CYP51, SQLE, and HMGCR was down-regulated during limonene biotransformation, indicating the inhibition of sterol biosynthesis (Table 3).

### Identification of the Differentially Expressed Genes Involved in Energy Metabolism

During limonene biotransformation, the DEGs correlated to energy metabolism were significantly down-regulated (Table 3). TCA cycle can generate metabolic intermediates and energy by a series of enzymes (Wang et al., 2019). In the present study, it is observed that most genes participated in TCA cycle were down-regulated after limonene induction, such as isocitrate dehydrogenase (IDH, PDIDSM_28150), succinate dehydrogenase (SDH, PDIDSM_53370), malate dehydrogenase (MDH, PDIDSM_47510), 2-oxoglutarate dehydrogenase (OGDH, PDIDSM_43060), phosphoenolpyruvate carboxykinase (pckA, PDIDSM_36580), pyruvate carboxylase (PDIDSM_16200), and so on. It implied that TCA cycle was blocked to reduce the production of energy for the growth and metabolism of *P. digitatum* DSM 62840 with limonene induction. It was probably because that *P. digitatum* was strongly inhibited by limonene. Limonene can be dissolved in the membrane cell because of its hydrophobicity, and this will result in the disruption of the membrane integrity and the increase of membrane permeability (Gonzalez-Estrada et al., 2018). The dysfunction of the membrane interfered the generation of ATP, which could inhibit the mycelia growth of the fungi (Sivakumar and Bautista-Banos, 2014). The present study also found that the weight of mycelia in P_12h was significantly higher than that in P_L_12h, and this showed that limonene inhibited the growth of *P. digitatum* (data not shown). In addition, the iTRAQ results revealed that the differentially expressed proteins related to energy metabolism were down-regulated during limonene transformation, such as NADH-ubiquinone oxidoreductase, SDH, and malic enzyme (Zhang et al., 2016). Beyond that, it is also observed that cytochrome C oxidase (CCO, PDIDSM_12860) and ubiquinol-cytochrome c reductase cytochrome b/c1 subunit (fbcH, PDIDSM_34810) involved in oxidative phosphorylation were down-regulated. CCO is a very important electron transporter which could transfer the electrons to generate H_2_O and ATP. The down-regulation of its expression could block electron transport chain, which will result in the incomplete oxidation and the overproduction of reactive oxygen species (Lin et al., 2020). It was speculated that limonene restrained the TCA pathway and inhibited the mycelial growth of *P. digitatum* cells via a membrane damage mechanism involving membrane peroxidation. Researchers have pointed out that terpene could damage the mitochondrial membrane permeability and disrupt the TCA pathway of *P. digitatum* (Zheng et al., 2015).

### Identification of the Differentially Expressed Genes Involved in ABC Transporters

As shown previously, limonene could inhibit the growth of the strain and have toxic effect on strain DSM 62840. When the monoterpenes entered into the cell, the fungi might activate an efflux pump system to reduce the toxicity of monoterpenes to the cells through the ABC transporters (Wang et al., 2013). ABC transporters are a large family of transmembrane proteins, and they can play an important role in the monoterpene resistance (Demissie et al., 2019). Wang et al. (2013) indicated that the transcript levels of ABC transporters were up-regulated when *Grosmannia clavigera* was exposed to monoterpenes. In the present study, most genes involved in ABC transports were observed to have a twofold increase in their transcript level after induction with limonene, including ABCB1 (PDIDSM_49740, PDIDSM_72790), ABCB6 (PDIDSM_719402), ABCC1 (PDIDSM_06070), FXA1/2 (PDIDSM_20430), PDR5 (PDIDSM_667602, PDIDSM_17720, PDIDSM_667603, PDIDSM_66760), and SNQ2 (PDIDSM_667608, PDIDSM_667609, PDIDSM_76180, PDIDSM_78490, PDIDSM_17650) (Table 3). Furthermore, the induction of limonene in transcript level was also supported by the results of iTRAQ analysis. The expression abundance of PDIDSM_667602, PDIDSM_719402, PDIDSM_17720, PDIDSM_76180 were significantly up-regulated by 2. 6-, 1. 3-, 2. 21-, and 5.06-fold with limonene induction, respectively (proteome data available via iProX partner repository with the dataset identifier PXD017752). It is hypothesized that the ABC transporters might identify and transport limonene to reduce the toxicity of limonene to strain DSM 62840.

### Identification of Candidate CYPs Involved in Limonene Biotransformation

Filamentous fungi cannot only secrete many primary and secondary metabolites, but also take part in a great deal of complex bioconversions, such as hydroxylation of alkanes, terpenes, fatty acids, flavonoids, sterols, as well as detoxication of xenobiotics. The mounting evidences have showed that CYPs may be responsible for these bioconversions. CYPs-dependent reactions have been proved to include hydroxylation, oxidation, epoxidation, dealkylation, dehydration, deamination, desaturation, dehalogenation, and demethylation (Makovec and Breskvar, 2000). In order to gain a better understanding on the enzymes which were involved in the limonene biotransformation, the influence of the inhibitors on the enzymatic activity, the enzyme localization, and the inducibility of this enzyme were assessed in this study.

**FIGURE 3:**
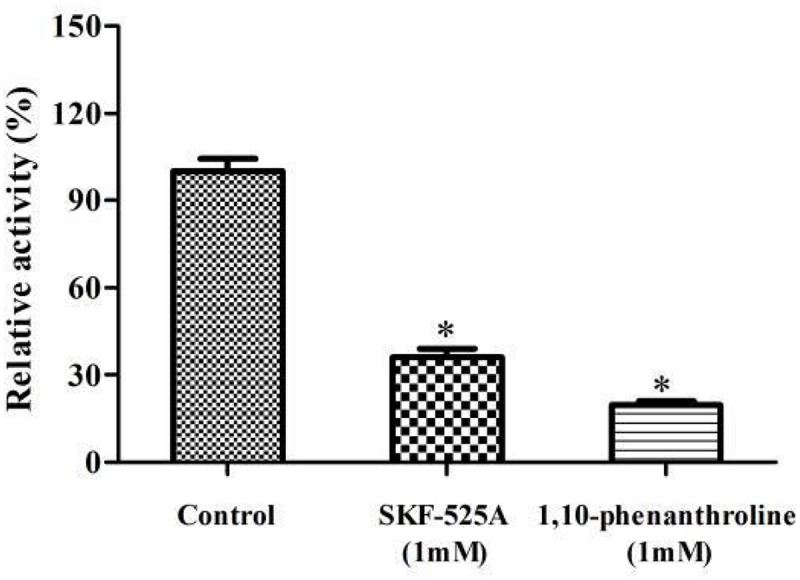
Effect of inhibitors on the enzymatic activity. The relative activity (%) was calculated using the control as 100%. Data were the mean ± SEM (*n* = 3). *p < 0.05 versus the control group.

The effect of the inhibitors on the enzymatic activity is shown in Figure 3. The presence of SKF-525A and 1,10-phenanthroline significantly inhibited the activity of the enzyme and retarded the limonene biotransformation (*p* < 0.05). SKF-525A is a well-known CYPs inhibitor. Earlier study clearly showed that the presence of SKF-525A inhibited the valencene transformation by *Chaetomium globosum*, suggesting the presence of CYPs during this transformation (Kaspera et al., 2005). Likewise, 1,10-phenanthroline is a metalloproteinase inhibitor and it could also significantly inhibit the limonene biotransformation. This might be due to the interaction between this inhibitor with the metal ion-active site of the enzyme. Tan et al. (1998) found that phenanthroline showed significant inhibitory effect on limonene bioconversion while the cyanide ions did not show this effect, and this implied that this catalysis system might also be CYPs.

**FIGURE 4:**
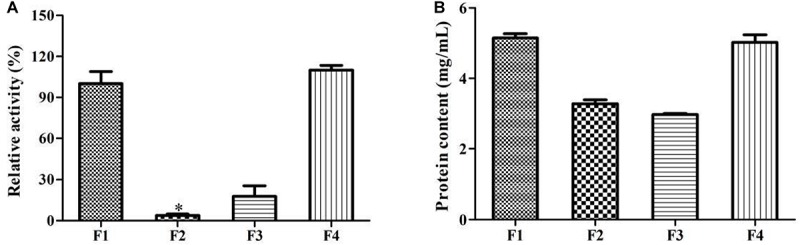
The experiments for the localization of enzyme involved in limonene biotransformation. **(A)** Comparison of the enzymatic activity from different components. **(B)** Comparison of the protein content from different components. F1, post-mitochondrial fractions; F2, cytosol fractions; F3, microsomal fractions; F4, microsomal fractions with 1% Triton X100 treatment. ^∗^*p* < 0.05 versus the F1 group. The relative activity (%) was calculated using the F1 as 100%. Data were the mean ± SEM (*n* = 3).

Interestingly, F1, F3, and F4 fractions were active while F2 was not active (Figure 4A). This indicated that the enzymes mainly existed in microsome. It is different from bacterial P450s that they are generally expressed as soluble enzymes, eukaryotic P450s are usually anchored to the inner mitochondrial or endoplasmic reticulum (microsomes). The components of microsomal P450s are membrane-associated proteins. They can be isolated in a microsomal fraction by ultracentrifugation (Bavishi et al., 2018). Triton X100 is a non-ionic detergent, and it could effectively destroy the membrane environment and promote the solubilization of membrane proteins. Our results clearly showed that the activity and protein content of F4 were higher than that of F3 (Figures 4A,B). Triton X100 treatment could increase the enzymes’ solubilization and activity. Earlier study implied that the α-terpineol dehydratase from *Pseudomonas gladioli* was a particulate-associated enzyme which can convert limonene to α-terpineol. It was partially solubilized in HEPES buffer which contained 2.0% Triton X100 (Cadwallader et al., 1992).

**FIGURE 5:**
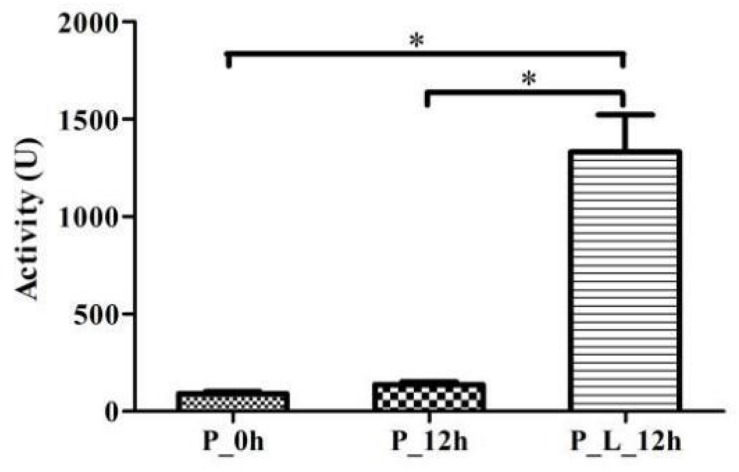
Effect of limonene induction on the enzymatic activity. P_0h, blank; P_12h, limonene-untreated blank; P_L_12h, limonene-treated condition. ^∗^*p* < 0.05. Data were the mean ± SEM (*n* = 3).

Additionally, it is found that the enzymatic activity of P_L_12h was significantly higher than that of P_0h and P_12h (*p* < 0.05), which were up to 13.72 and 8.84%, respectively (Figure 5). It demonstrated that the enzyme involved in this biotransformation was inducible. Prieto et al. (2011) also indicated that the enzyme which was responsible for the limonene oxyfunctionalization was inducible in *P. digitatum* DSM 62840. In contrast, the enzyme from *Sphingobium* sp. was cofactor independent, non-inducible and was able to generate α-terpineol from limonene. This enzyme preferred anaerobic conditions during the biotransformation, and this indicated that it was a hydratase rather than cytochrome P450 (Bicas et al., 2010).

Based on the above results, we had reasons to believe that membrane-associated cytochrome P450 enzymes might be responsible for the limonene biotransformation. In order to find the CYPs genes and pathways related to limonene biotransformation, these two following conditions of the selected genes were required: (1) the genes were assigned to the fungal cytochrome P450 database; (2) the genes were differentially expressed (*P*-value ≤ 0.05, twofold threshold) in both “P_0h vs. P_L_12h” and “P_12h vs. P_L_12h.” These results showed that a total of 58 DEGs were obtained, of which 31 were up-regulated and 27 were down-regulated (Supplementary Table S7). It was possible that some of these potential CYPs were involved in the limonene metabolism (Table 3). Among these potential candidate genes, the highest expression level was observed for PDIDSM_85260, which was highly similar to the fungal transcriptional regulatory protein in *Penicillium camemberti* FM013 at the protein level (94% identity and 95% similarity). PDIDSM_85260 was significantly up-regulated by 3,640- and 3,258-fold in “P_0h vs. P_L_12h” and “P_12h vs. P_L_12h,” respectively. And it was annotated as hypothetical protein PDIG_38620 from *P. digitatum* PHI26 containing a GAL4-like Zn2Cys6 binuclear cluster DNA-binding domain and a fungal transcription factor regulatory middle homology region, suggesting a possible function as a transcription factor. Most of the zinc proteins participated either in the utilization of carbon/nitrogen sources, or the biosynthesis of amino acids/secondary metabolites, such as MlcR which was involved in the biosynthesis of compactin in *Penicillium citrinum* (Baba et al., 2008), AflR which participated in the biosynthesis of toxic aflatoxin in *Aspergillus flavus* (Woloshuk et al., 1994), NirA which was related to nitrate utilization in *Aspergillus nidulans* (Burger et al., 1991), as well as Gal4 which was responsible for galactose metabolism in *Saccharomyces cerevisiae* (Traven et al., 2006). Thus, the high expression of PDIDSM_85260 suggested that it might take an important part in limonene bioconversion. Limonene was utilized and could activate the transcription of PDIDSM_85260 genes during this biotransformation. However, the actual function and role of PDIDSM_85260 remained to be further explored.

To our knowledge, CYPs belong to a superfamily of heme-containing proteins which play a central role in oxidation of xenobiotics and endogenous compounds (Buathong et al., 2019). CYPs catalyze the insertion of one atom of molecular oxygen into substrates while the other atom is reduced to water. The oxidation of substrates via CYPs requires electron transfer partner, such as a two-protein system (ferredoxin and ferredoxin reductase) for bacterial P450s, or NADPH-dependent cytochrome P450 reductase (CPR) for eukaryotic microsomal P450s. Almost all eukaryotic P450s require a protein partner CPR to gain electrons from NADPH for oxygen activation. CPR belongs to the diflavin reductase protein family, and it is located in microsomes (Ji et al., 2019). In the present study, the expression level of PDIDSM_67430 encoding CPR was strongly stimulated by limonene. CPR expression was increased approximately 132- and 117-fold in P_L_12h compared with that in P_0h and P_12h, respectively. The up-regulation of CPR expression suggested that it might take an important role in limonene biotransformation. During the oxidation reaction of limonene catalyzed CYPs, an oxygen molecule was activated and one of its oxygen atoms was inserted into limonene, the other one was reduced to a water molecule. Meanwhile, the corresponding electron transfer partner CPR was simultaneously induced. The electrons were derived from NADPH and shuttled through CPR into the central heme-group of the CYP. Unfortunately, CPR protein was not found in the proteome data. This may be attributable to the differences of the operating conditions, the surrounding environment, and the samples from different batches, etc. And it has been reported that gene expressions at a transcription could not always transduce up to the protein synthesis due to the complicated post-transcriptional mechanisms (Greenbaum et al., 2003).

Further analysis showed that CPR was involved in tryptophan metabolism and fatty acid degradation pathways and was responsible for the hydroxylation of melatonin and fatty acid according to KEGG annotation. The enzyme catalyzing the hydroxylation of melatonin to 6-hydroxymelatonin is localized in the microsomes and requires molecular oxygen and NADPH. CYP1A1, CYP1A2, and CYP1B1 are the important catalyst of the melatonin hydroxylation, of which CYP1A2 is the principal part (Facciola et al., 2001; Skene et al., 2001; Foster et al., 2015). In addition, we noted that several genes were also associated with the hydroxylation of melatonin and fatty acid, including hypothetical protein PDIDSM_86880, PDIDSM_67660 encoding cytochrome P450 alkane hydroxylase, PDIDSM_67750 encoding cytochrome P450 monooxygenas, and PDIDSM_08220 encoding P450 family fatty acid hydroxylase (CYP102A2_3). Among these proteins, the PDIDSM_86880 was related to T1PKS-terpene clusters and assigned to iron ion binding, oxidoreductase activity, heme binding and oxidation-reduction process term by GO annotation. There was 89% sequence identity between PDIDSM_86880 and cytochrome P450 from *Penicillium expansum* at amino acid level, suggesting that PDIDSM_86880 might possess a putative cytochrome P450 activity. The PDIDSM_86880 was significantly up-regulated in “P_0h vs. P_L_12h” and “P_12h vs. P_L_12h” (9.9- and 6.4-fold, respectively) when incubating with limonene, suggesting that it might be likely involved in the biotransformation.

Previously, CYP102 family members have been proven to be capable of hydroxylating fatty acids (Munday et al., 2016). Of those, CYP102A1 (P450 BM3), a high activity fatty acid hydroxylase, is the first and the most representative member. It has been widely regarded as a model for studying the mechanisms of different substrates hydroxylation, such as sterols, flavonoids, and terpenes. It is able to produce perillyl alcohol from limonene, and its variations VVF (A264 V/A328 V/L437F) could increase the regioselectivity of limonene C-7 oxidation by up to 97% (Kim and de Montellano, 2009). Furthermore, other CYP102s have been confirmed that they share many properties with CYP102A1, such as substrate specificity. CYP102A7 was not only involved in fatty acid hydroxylation, but also exhibited hydroxylation activity for terpenes and terpenoids. Specifically, the main product of limonene oxidation was 1, 2-limonene epoxide, carveol and 8, 9-limonene epoxide by CYP102A7 (Dietrich et al., 2008). Limonene-8,9-epoxide has been implicated as an intermediate in limonene bioconversion. It was hypothesized that α-terpineol was obtained from limonene via epoxidation of limonene and reductive cleavage of epoxide using *P. digitatum* (Tan et al., 1998). Hence, we speculated that CYP102A2_3 from strain DSM 62840 might have the similar catalytic function with the CYP102A7, and it might catalyze limonene to limonene-8,9-epoxide, which would result in the production of α-terpineol. Regretfully, CYP102A2_3 expression was significantly down-regulated in “P_0h vs. P_L_12h” and “P_12h vs. P_L_12h” (3.3- and 5.1-fold, respectively) when this fungal was incubated with limonene in the present study. Gene expression levels can be affected by a variety of factors. Further studies are necessary to verify the functions of CYP102A2_3.

In addition, some genes were predicted to possess benzoate 4-monooxygenase (CYP53A15) activity, such as PDIDSM_577403, PDIDSM_42610, PDIDSM_12990, PDIDSM_0468015, PDIDSM_62110, and so on. Interesting, these genes were up-regulated in both “P_0h vs. P_L_12h” and “P_12h vs. P_L_12h.” CYP53A15 belongs to CYP53 family that is highly conserved proteins. CYP53A15 is correlated to detoxification and degradation of benzoate, and is capable of hydroxylating benzoate to 4-hydroxybenzoate. It can be further metabolized by the β-ketoadipate pathway to generate intermediates which will enter into the TCA cycle (Tamaki et al., 2018). Benzoate is essential for the metabolism pathways of aromatic compounds, and could inhibit fungal growth. It is reported that benzoate 4-monooxygenase gene from *Cochliobolus heterostrophus* was up-regulated after the infection of maize, and this demonstrated that benzoate detoxification in plant pathogenic fungi contributed to their pathogenicity (Degani, 2014). In addition, the proteomic studies showed a significant rise in PDIDSM_62110 with limonene treatment, which was in agreement with the findings of the transcriptome data. It is inferred that limonene transformation may be associated with the detoxification and degradation of benzoate. Further efforts are needed to confirm this assumption.

### Validation of RNA-Seq Results by RT-qPCR

**FIGURE 6:**
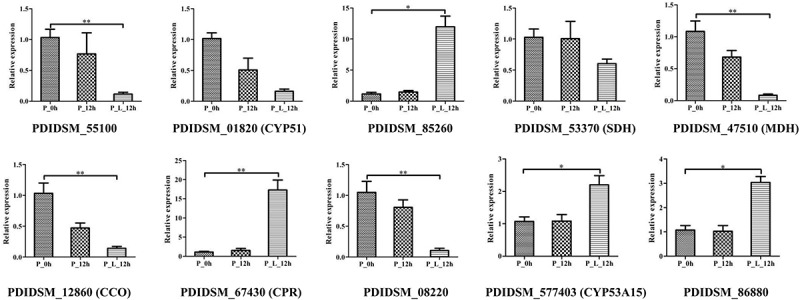
The verification of relative expression of genes by quantitative real-time polymerase chain reaction (RT-qPCR). CYP51, sterol 14-demethylase; SDH, succinate dehydrogenase; MDH, malate dehydrogenase; CCO, cytochrome C oxidase; CPR, NADPH-dependent cytochrome P450 reductase; CYP53A15, benzoate 4-monooxygenase; P_0h, blank; P_12h, limonene-untreated blank; P_L_12h, limonene-treated condition, ^∗^*p* < 0.05, ^∗∗^*p* < 0.01.

Ten DEGs were selected for RT-qPCR analysis to validate the transcriptome data (Figure 6). The results showed that the expression level of CYP51 and SDH decreased with limonene treatment, and PDIDSM_55100 (*p* < 0.01), MDH (*p* < 0.01), CCO (*p* < 0.01), and PDIDSM_08220 (*p* < 0.01) were significantly down-regulated with limonene treatment. In contrast, the transcript abundance of CPR (*p* < 0.01), CYP53A15 (*p* < 0.01), PDIDSM_86880 (*p* < 0.05), and PDIDSM_85260 (*p* < 0.01) was significantly up-regulated in P_L_12h compared with that in P_0h. This result manifested that RT-qPCR data gave good agreement with the transcriptome data.

## Conclusion

In the present study, the molecular mechanisms of limonene bioconversion in *P. digitatum* DSM 62840 were analyzed and discussed by using genomic and transcriptomic analysis. The strain DSM 62840 genome was estimated to be 29.09 Mb and encoded 9,086 protein-encoding genes. And the most annotated genes were associated to some protein metabolism and energy metabolism functions. The transcriptome analysis revealed that a total of 4,128 and 4,148 DEGs (≥2-fold change) were identified in both “P_0h vs. P_L_12h” and “P_12h vs. P_L_12h.” These DEGs were involved in metabolic pathways, biosynthesis of antibiotics, biosynthesis of amino acids, carbon metabolism, ribosome, biosynthesis of secondary metabolites. Among these DEGs, the genes involved in TCA cycle and oxidative phosphorylation were down-regulated during this bioconversion, and the genes involved in ABC transporters terms were up-regulated. On the other hand, it is demonstrated that the enzyme involved in this biotransformation was inducible, and it was located in the microsomes and was obviously inhibited by the CYP inhibitors. This suggested that CYP might play a role in limonene biotransformation. It was further observed that several differentially expressed cytochrome P450 genes, such as PDIDSM_85260 and PDIDSM_67430, were significantly up-regulated during limonene biotransformation. It is speculated that these genes may participate in the biotransformation of limonene to α-terpineol. This study improved our understanding on the mechanism of the bioconversion of limonene to α-terpineol, and provided a series of candidate genes for the further research.

## Data Availability Statement

The genomic data produced for this article were deposited at DDBJ/ENA/GenBank under the accession JAAQRF000000000. The clean reads have been deposited at NCBI’s Sequence Read Archive (SRA) under accession numbers PRJNA608770. The RNA-seq data were available in the NCBI SRA database under accession number PRJNA608617.

## Author Contributions

GF and JH conceived and designed the study. Y-YZ, ZL, and J-NR performed the experiments. WH, XL, and S-YP analyzed the data. L-LZ wrote and edited the manuscript. L-LZ drew the image.

## Conflict of Interest

The authors declare that the research was conducted in the absence of any commercial or financial relationships that could be construed as a potential conflict of interest.

## Footnotes

1 https://www.sigmaaldrich.com/china-mainland.html

2 https://github.com/BGI-flexlab/SOAPnuke

3 https://www.ncbi.nlm.nih.gov/genome
